# Synthesis of PtM (M=Co, Ni)/Reduced Graphene Oxide Nanocomposites as Electrocatalysts for the Oxygen Reduction Reaction

**DOI:** 10.1186/s11671-015-1208-5

**Published:** 2016-01-05

**Authors:** Jialiang Li, Xinning Fu, Zhou Mao, Yushi Yang, Tong Qiu, Qingzhi Wu

**Affiliations:** School of Chemical Engineering, Shandong University of Technology, Zibo, 255049 China; State Key Laboratory of Advanced Technology for Materials Synthesis and Processing, and Biomedical Material and Engineering Center, Wuhan University of Technology, Wuhan, 430070 China

**Keywords:** Pt-based alloy, Reduced graphene oxide, Oxygen reduction reaction

## Abstract

A series of PtM (M=Co, Ni)/reduced graphene oxide (rG-O) nanocomposites were successfully synthesized through a facile hydrothermal method. The as-synthesized nanocomposites were characterized using transmission electron microscopy and high-resolution transmission electron microscopy, X-ray diffraction, inductively coupled plasma-atomic emission spectrometer, and X-ray photoelectron spectroscopy. The electrochemical performance and oxygen reduction reaction (ORR) activity of PtM/rG-O nanocomposites were evaluated using cyclic voltammetry and the rotating disk electrode method. The results show that the addition of the reductant (1,2-hexadecanediol, HAD) in the reaction system slightly improved the ORR activity of PtM/rG-O nanocomposites with a negligible influence on the size and morphology of alloy NPs. Furthermore, PtNi/rG-O nanocomposites displayed the higher electrochemical stability than PtCo/rG-O nanocomposites. These results provide a facile strategy for the synthesis of Pt-based alloy NPs/rG-O nanocomposites for applications in catalysis and energy-related processes.

## Background

Noble metal platinum (Pt) is considered as one of the most important catalysts in modern industry (such as chemical synthesis and fuel cell) due to its rather high catalytic activity [1, 2]. Nevertheless, the extensive application of Pt catalyst is seriously limited because of its high cost and easy deactivation from the surface adsorption of poisonous intermediates or reaction products. One of the solutions to overcome these drawbacks is to explore Pt-based alloys with low-cost *3d* transition metals (such as Fe, Co, Ni, and Cu) [3–6]. The presence of *3d* transition metals is favorable to minimize the adsorption of poisonous intermediates or reaction products on the surface of the catalyst, resulting in the increase of the active sites for reactant molecules [7, 8].

Graphene is a promising matrix for catalysts because of its electrical and thermal conductivity, mechanical properties, and high specific surface area [9]. Various metal oxides (such as TiO_2_, Fe_3_O_4_, and Co_3_O_4_) [10–12] and noble metals (such as Pt and Pd) [13–16] have been loaded on the surface of reduced graphene oxide (rG-O), which displayed the improved catalytic activity on a series of reactions, such as the ORR, oxygen evolution reaction, and degradation of organic dyes.

In this work, PtM (M=Co, Ni)/rG-O nanocomposites were synthesized through a facile hydrothermal route. The influence of the reductant (1,2-hexadecanediol, HAD) on the size and shape of PtM NPs was studied. Furthermore, the electrochemical performance and ORR activity of PtM/rG-O nanocomposites were evaluated using cyclic voltammetry (CV) and the rotating disk electrode (RDE) method.

## Methods

### Reagents

Platinum acetylacetonate (Pt(acac)_2_, 97 %) was from Sigma-Aldrich Corp., St Louis, MO. Other chemicals were of analytical grade (Sinopharm Chemical Reagent Co., Ltd) and used without further purification. Deionized water (16 MΩ · cm) was obtained from a Nanopure Water Systems UV (Thomas Scientific, Swedesboro, NJ).

### Synthesis of PtM/rG-O Nanocomposites

GO was prepared using natural graphite powder (Sinopharm Chemical Reagent Co., Ltd) according to the modified Hummer’s method. Prior to the synthesis of PtM/rG-O nanocomposites, the as-prepared GO was dispersed in deionized water by ultrasonication (KQ2200E system, Kunshan Ultrasonic Instruments Co., Ltd, 40 KHz, 80 W) for 3 h. PtM/rG-O nanocomposites were synthesized by the solvothermal method using ethylene glycol (EG)-water as the solvent. In a typical synthesis, Pt(acac)_2_ (0.25 mmol, 0.0985 g) was dissolved in EG (30 mL) under magnetic stirring with a short heating (90–100 °C, 5 min). Co(NO_3_)_2_⋅6H_2_O (0.25 mmol, 0.0728 g) or NiSO_4_⋅6H_2_O (0.25 mmol, 0.0657 g) was subsequently dissolved in the solution containing Pt(acac)_2_. In the presence of the additional reductant, 1,2-hexadecanediol (HAD, 0.5 mmol, 0.129 g) was dissolved in EG (10 mL) and then added dropwise in the EG solution containing the metal salts (20 mL). Then, 10 mL of GO aqueous solution (2 mg/mL) was added dropwise into the EG solution. After 30 min of stirring, the mixture was transferred to, and sealed in, a 50-mL Teflon-lined stainless steel autoclave and heated to 180 °C for 8 h and then cooled to room temperature. The precipitate was collected and washed alternately with ethanol and deionized water by centrifugation (10,000 rpm, 5 min) and then dried at 60 °C in vacuum.

### Characterizations of PtM/rG-O Nanocomposites

The phase structure of the samples was characterized by X-ray diffraction (XRD; D/MAX-RB, RIGAKU Corp., Japan) using Cu Kα radiation (*λ*) 1.5406 Å. The morphology of the samples was observed by transmission electron microscopy (TEM, Tecnai G2 20, FEI Corp., the Netherlands) and high-resolution transmission electron microscopy (HRTEM, JEM-2100 F, JEOL Corp., Japan). X-ray photoelectron spectroscopy (XPS) was done on a VG Multilab 2000 (Thermo Electron Corp., MA) using Al Kα radiation as the excitation source. Raman spectra were recorded using a micro-Raman spectrometer (INVIA, RENISHAW Corp.; 785-nm excitation wavelength). The elemental composition was obtained with an Optima 4300DV inductively coupled plasma-atomic emission spectrometer (ICP-AES) (Optima 4300DV, PerkinElmer Corp.).

### Electrochemical Measurement

The electrochemical performance of PtM/rG-O nanocomposites was measured with a conventional triple electrode system. A saturated calomel electrode (SCE) was used as the reference electrode and a platinum foil as the counter electrode. The sample was mixed with 1500.0 μL of deionized water, 400.0 μL of isopropanol, and 100.0 μL of Nafion solution (0.5 wt.%) to form a 2-mg/mL suspension, then 5 μL of ink was dispersed onto a mirror-polished glassy carbon disk electrode (*f* = 5 mm) as the working electrode. CV curves were obtained in 0.1 M HClO_4_ solution at the scan rate of 50 mV/s at room temperature in a potential window of 0–1.2 V versus the reversible hydrogen electrode (RHE). The electrochemical active surface area (ECSA) was calculated based on the formula ECSA = *Q*_*H*_/(*m*_Pt_ × *q*_*H*_), where *Q*_*H*_ is the charge for *H*_upd_ adsorption, *m*_Pt_ is the loading amount of metal, and *q*_*H*_ (210 μC cm^−2^) is the charge required for monolayer adsorption of hydrogen on Pt surfaces [17]. The ORR activity of different samples was evaluated by the rotating disk electrode (RDE) technique in O_2_-saturated 0.1 M HClO_4_ solution with a sweep rate of 10 mV s^−1^ at 1600 rpm, at room temperature.

## Results and Discussion

**Fig. 1:**
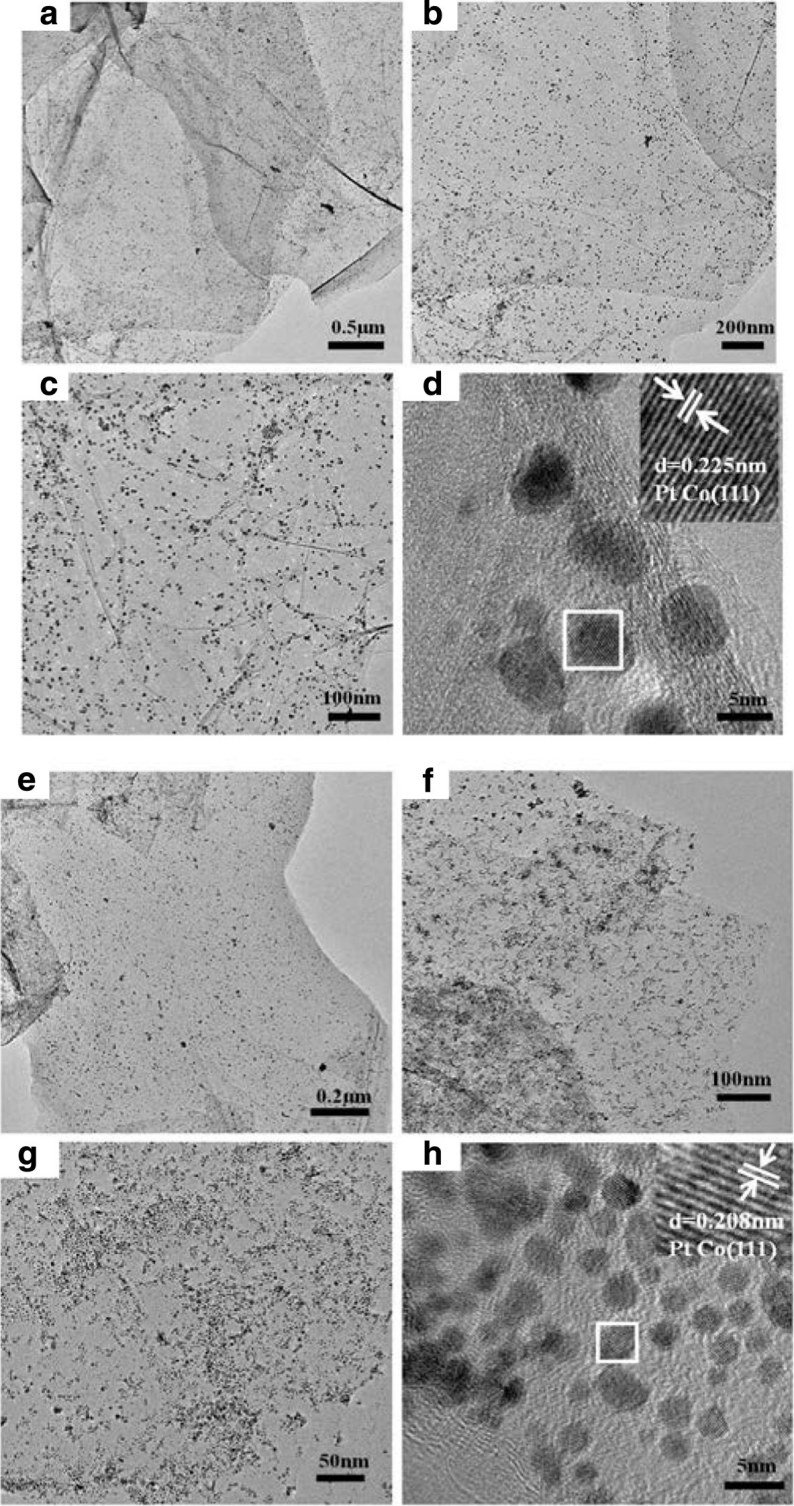
TEM and HRTEM images of PtCo/rG-O nanocomposites synthesized in the absence and presence of HAD. **a**–**d** PtCo/rG-O; **e**–**h** PtCo/rG-O-HAD

Figure 1 shows TEM and HRTEM images of PtCo/rG-O nanocomposites synthesized with and without addition of the reductant (HAD). Single-layer rG-O sheets in a large area were observed, and monodisperse PtCo alloy NPs were homogeneously loaded on the surface of rG-O sheets. PtCo NPs are roughly spherical with an average size of ca. 4.0 and 3.0 nm, corresponding to the absence and presence of HAD. The insets in Fig. 1d, h display the well-aligned lattice planes, indicating the single crystalline nature of PtCo in both of the samples. The interplanar spacing of ca. 0.225 and 0.208 nm obtained from the HRTEM image could be indexed to the (111) plane of PtCo.

**Fig. 2:**
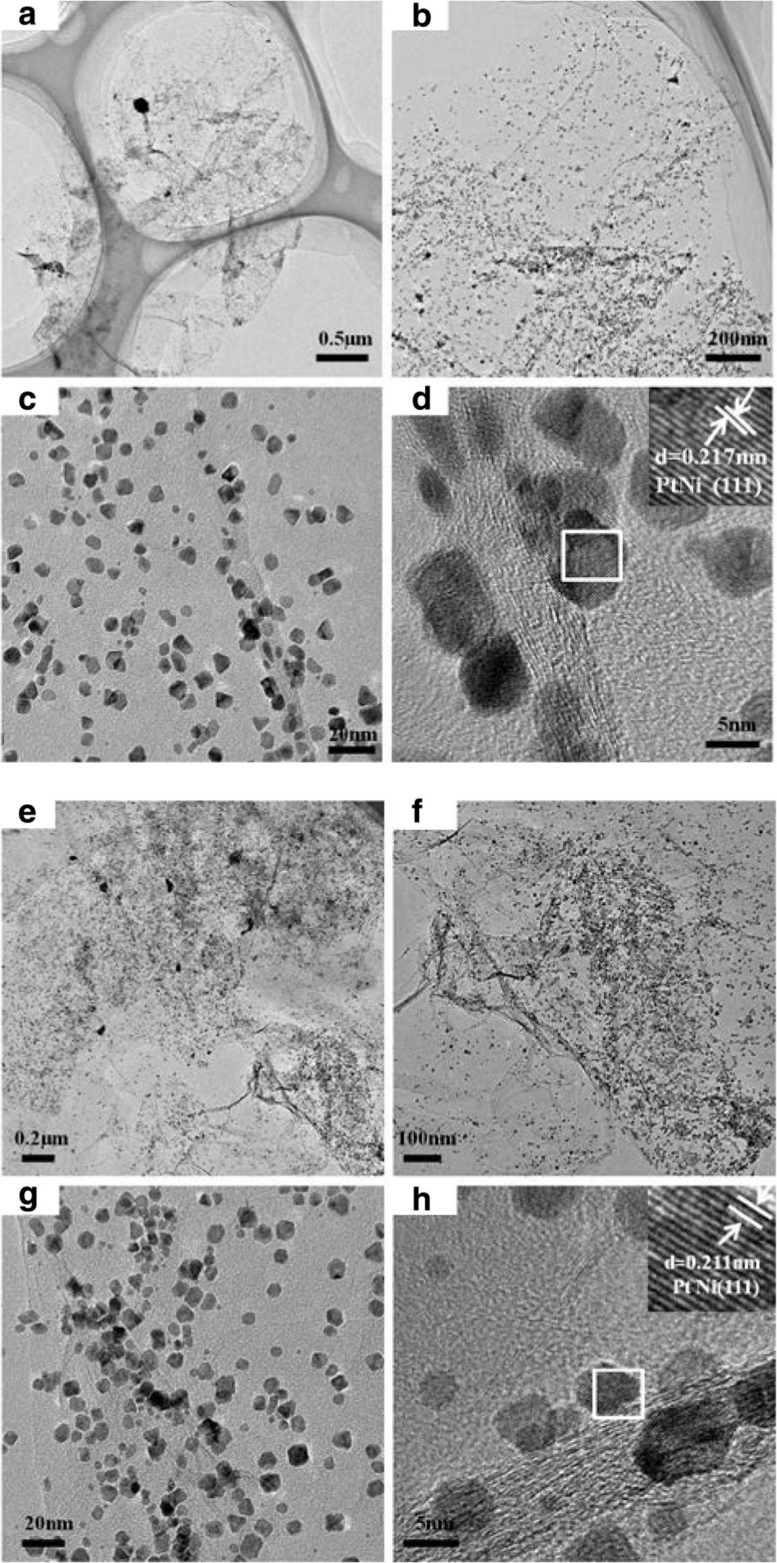
TEM and HRTEM images of PtNi/rG-O nanocomposites synthesized in the absence and presence of HAD. **a**–**d** PtNi/rG-O; **e**–**h** PtNi/rG-O-HAD

By comparison, PtNi nanocubes with an average size of ca. 4.0 nm were obtained in the presence of HAD, instead of the irregular shape with an average size of 4.5 nm in the absence of HAD, as shown in Fig. 2. The well-aligned lattice planes in the insets of Fig. 2d, h indicate the single crystalline nature of PtNi alloy. The interplanar spacing of ca. 0.217 and 0.211 nm obtained from the HRTEM image could be indexed to the (111) plane of PtNi. It is noticeable that the interplanar spacing in both PtCo and PtNi NPs is smaller than that in pure Pt (0.227 nm), implying the successful incorporation of Co and Ni atoms into the lattices of Pt [18]. Meanwhile, the addition of the reductant (HAD) promoted the reduction of Co and Ni and thus increased the ratio of Co and Ni in alloy NPs, which resulted in the further contraction of the lattice [19, 20].

The composition of as-synthesized PtM/rG-O nanocomposites was analyzed by ICP-AES. The results suggested an average composition of Pt_67_Co_33_ for PtCo/rG-O, Pt_62_Co_38_ for PtCo/rG-O-HAD, Pt_74_Ni_26_ for PtNi/rG-O, and Pt_69_Ni_31_ for PtNi/rG-O-HAD. These results suggested that the addition of HAD slightly decreased the size of PtM alloy NPs and improved the component of transition metals. The low ratio of Co and Ni in alloy NPs could be attributed to the weak reducing ability of the reaction system, which resulted in the partial reduction of Co^2+^ and Ni^2+^ into Co^0^ and Ni^0^ [21].

**Fig. 3:**
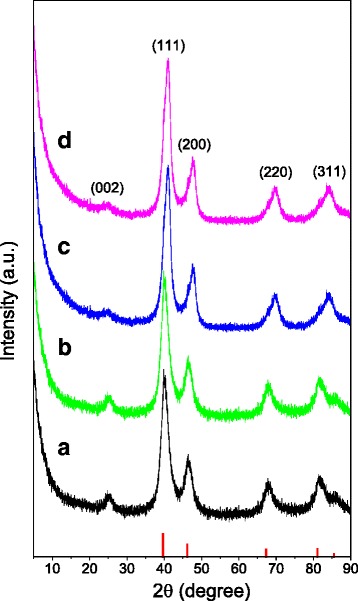
XRD patterns of the as-synthesized PtM/rG-O nanocomposites. **a** PtCo/rG-O; **b** PtCo/rG-O-HAD; **c** PtNi/rG-O; **d** PtNi/rG-O-HAD

The phase structure of the as-synthesized PtM/rG-O nanocomposites was identified by XRD, as shown in Fig. 3. Compared with pure Pt (JCPDS card no. 04-0802), a slight shift toward the higher angle was observed in all characteristic peaks of PtM/rG-O nanocomposites, indicating that the introduction of Co and Ni modified the crystal structure [22, 23]. The peaks in XRD patterns of PtM/rG-O nanocomposites were indexed to the (111), (200), (220), and (311) planes of PtCo and PtNi, respectively. The peak at ca. 23.5° (2*θ*) was assigned as the (002) carbon peak of rG-O, implying the successful reduction of GO to rG-O [24, 25]. No diffraction peaks derived from cobalt oxides and nickel oxides were observed in the XRD patterns. In addition, the average size calculated according to Scherrer’s formula is 3.9 nm for PtCo/rG-O, 2.6 nm for PtCo/rG-O-HAD, 4.1 nm for PtNi/rG-O, and 3.5 nm for PtNi/rG-O-HAD, which are in reasonable agreement with that from TEM images.

**Fig. 4:**
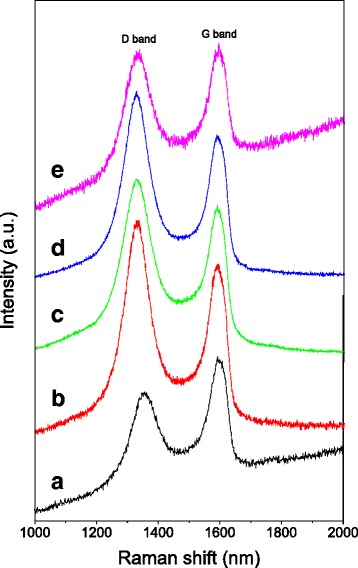
Raman spectra of PtM/rG-O nanocomposites. **a** GO; **b** PtCo/rG-O; **c** PtCo/rG-O-HAD; **d** PtNi/rG-O; **e** PtNi/rG-O-HAD

Figure 4 shows the Raman spectra of GO and PtM/rG-O nanocomposites. The characteristic peak at ca. 1585 cm^−1^ (G band) was from the in-plane vibration of symmetric *sp*^*2*^ C-C bonds, whereas the peak at ca. 1350 (D band) was from the first-order zone boundary phonon [26]. The intensity ratio between the D band and G band (*I*_D_/*I*_G_) was 1.21 for PtCo/rG-O, 1.15 for PtCo/rG-O-HAD, 1.23 for PtNi/rG-O, and 1.01 for PtNi/rG-O-HAD, which are larger than that of GO (0.85). The increased *I*_D_/*I*_G_ ratio is indicative of a decrease in the average size of *sp*^*2*^ domains owing to the reduction process [27].

**Fig. 5:**
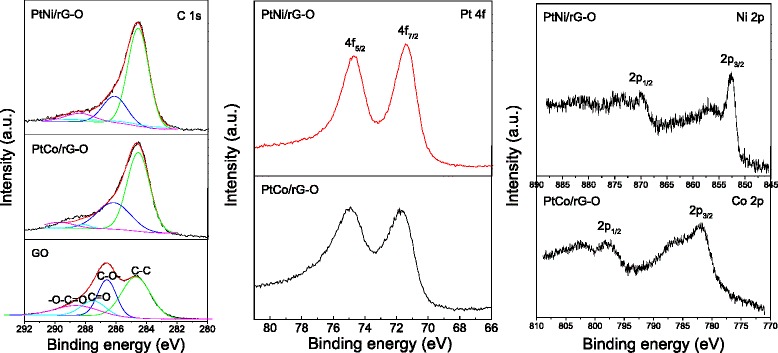
XPS spectra of C 1s, Pt 4f, Co 2p, and Ni 2p in PtM/rG-O-HAD nanocomposites

The chemical states of C, Pt, Co, and Ni elements in the as-synthesized PtM/rG-O nanocomposites were characterized by XPS. Figure 5 shows XPS spectra of C 1s, Pt 4f, Co 2p, and Ni 2p in PtM/rG-O-HAD (The similar XPS spectra derived from PtM/rG-O were not shown). As shown in XPS spectra of C 1s in GO and PtM/rG-O-HAD, four different carbon bonding states were fitted and identified. The peak at 284.5 eV was attributed to the C-C bond, whereas the peaks at ca. 286.6, 287.7, and 289 eV were assigned to the epoxy, carbonyl, and carboxylate bond, respectively. The high signal intensity derived from the C-O band indicated the high oxidation degree in GO. By comparison, the peaks derived from the C-O band significantly decreased in XPS spectra of PtM/rG-O-HAD, implying the loss of oxygen species and the reduction of GO into rG-O. The XPS spectra of Pt 4f in PtM/rG-O-HAD showed two spin-orbit doublets with maxima at ca. 71.8 eV (Pt 4f_7/2_) and 75.1 eV (Pt 4f_5/2_) for PtCo/rG-O-HAD and at ca. 71.5 eV (Pt 4f_7/2_) and 74.7 eV (Pt 4f_5/2_) for PtNi/rG-O-HAD, which are slightly higher than those of pure Pt (71.4 eV for Pt 4f_7/2_ and 74.8 eV for Pt 4f_5/2_). This shift could be attributed to the formation of PtM alloy. In XPS spectra of Co 2p and Ni 2p, several satellite peaks were observed except of the peaks derived from the zero-valent Co 2p and Ni 2p, which could be attributed to the slight oxidation of Co and Ni atoms on the surface of alloy NPs, as well as the bonding of alloy NPs with the functional groups (such as -OH and O-C=O) on rG-O sheets.

**Fig. 6:**
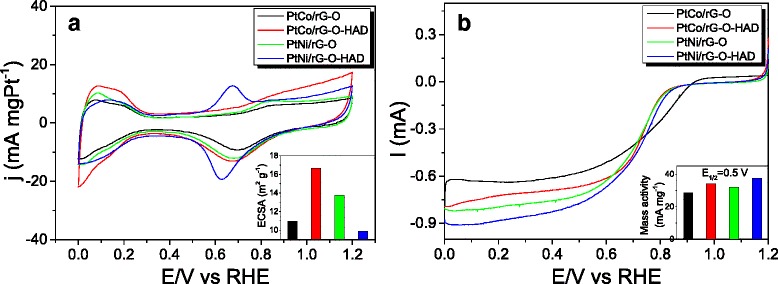
Electrochemical performance of PtM/rG-O nanocomposites. **a** CV curves of PtM/rG-O nanocomposites measured in 0.1 M HClO_4_ solution at a scan rate of 50 mV s^−1^ at room temperature; **b** ORR activities of PtM/rG-O nanocomposites measured at a speed of 1600 rpm in O_2_-saturated 0.1 M HClO_4_ solution at room temperature. The *inset* in **a** and **b** is the ECSA value and mass activity (*E*_1/2_ = 0.5) of PtM/rG-O nanocomposites, respectively

The electrochemical performance of the as-synthesized PtM/rG-O nanocomposites was evaluated by CV in 0.1 M HClO_4_ solution at a scan rate of 50 mV s^−1^. As shown in Fig. 6a, all CV curves exhibited three characteristic potential regions: the hydrogen under-potential adsorption/desorption (H-UPD) region, the double-layer region, and the metal oxidation/reduction region. The ECSA value of PtM/rG-O based on Pt mass was calculated using the H-UPD method [17]. As shown in the inset of Fig. 6a, the ECSA value was ca. 11.0 m^2^g^−1^ for PtCo/rG-O, 16.6 m^2^g^−1^ for PtCo/rG-O-HAD, 13.7 m^2^g^−1^ for PtNi/rG-O, and 9.9 m^2^g^−1^ for PtNi/rG-O-HAD. The ECSA value of the PtM/rG-O nanocomposites was obviously lower than that of the commercial Pt/C catalyst (approximately 33–55 m^2^/g Pt) [28–30], which may be attributed to the composition and crystalline structure, and the loading density of alloy NPs. More investigations are carried out in order to explore the detailed mechanisms for the decreased electrocatalytic performance of PtM/rG-O nanocomposites.

The ORR activity of the as-synthesized PtM/rG-O nanocomposites was also measured using an RDE operated at 1600 rpm in O_2_-saturated 0.1 M HClO_4_. Figure 6b shows the polarization curves of PtM/rG-O nanocomposites. The electrocatalytic activity was estimated from the half-wave potentials (*E*_1/2_) versus the RHE. The maximum *E*_1/2_ value was 0.93 V for PtCo/rG-O, 1.08 V for PtCo/rG-O-HAD, 1.14 V for PtNi/rG-O, and 1.08 V for PtNi/rG-O-HAD. Furthermore, the mass-specific kinetic currents (mass specific activity) of the different samples were calculated according to the Levich-Koutecky equation [31]:

$$ 1/i=1/{i}_k+1/{i}_d $$

where *i* is the experimentally measured current, *i*_*d*_ is the diffusion-limiting current, and *i*_*k*_ is the kinetic current. As shown in the inset of Fig. 6b, the mass-specific activity at *E*_1/2_ = 0.5 V is 32 mA/mg Pt for PtCo/rG-O, 37.4 mA/mg Pt for PtCo/rG-O-HAD, 28.6 mA/mg Pt for PtNi/rG-O, and 34.0 mA/mg Pt for PtNi/rG-O-HAD. These results show that the enhanced reduction ability of the reaction system may promote the mass-specific activity of PtM/rG-O nanocomposites.

**Fig. 7:**
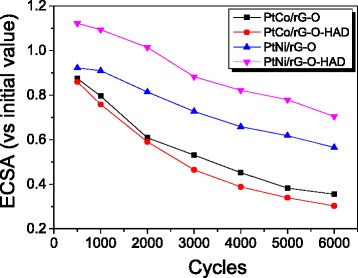
Electrochemical stability of PtM/rG-O nanocomposites after 6000 cycles

To evaluate the stability of the as-synthesized PtM/rG-O nanocomposites, potential sweeps between 0 and 1.2 V were performed for 6000 cycles in 0.1 M HClO_4_ solution at room temperature. The ECSA values of the as-synthesized PtM/rG-O nanocomposites calculated from their CVs were plotted as a function of the cycle number (Fig. 7). The loss of ECSA value was ca. 65–70 % for PtCo/rG-O nanocomposites and ca. 30–45 % for PtNi/rG-O nanocomposites after 6000 cycles, indicating the significantly higher stability of PtNi/rG-O than that of PtCo/rG-O with and without addition of HAD.

## Conclusions

In summary, PtM (M=Co, Ni)/rG-O nanocomposites were successfully synthesized through a facile hydrothermal route. PtM alloy NPs were homogenously loaded on the surface of rG-O sheets. The results show that the addition of the reductant (HAD) in the reaction system slightly improved the ORR activity of PtM/rG-O, which displayed a negligible influence on the size and shape of alloy NPs. Meanwhile, PtNi/rG-O nanocomposites displayed the higher electrochemical stability than PtCo/rG-O nanocomposites. These results provide a facile strategy for the synthesis of various Pt-based alloy/rG-O nanocomposites for applications in catalysis and energy-related processes.
